# The role of SMAD signaling in hypertrophic obstructive cardiomyopathy: an immunohistopathological study in pediatric and adult patients

**DOI:** 10.1038/s41598-023-30776-9

**Published:** 2023-03-06

**Authors:** Zhengjie Zhang, Fengzhi Zhang, Mingkui Zhang, Hui Xue, Lixin Fan, Yan Weng

**Affiliations:** 1 First Hospital of Tsinghua University, Beijing, 100016 China; 2 Institute for Precision Medicine, Tsinghua University, Beijing, 100084 China

**Keywords:** Cardiovascular biology, Cardiovascular diseases

## Abstract

Hypertrophic obstructive cardiomyopathy (HOCM) can bring a high risk of sudden cardiac death in young people. It is particularly urgent to understand the development and mechanism of HOCM to prevent unsafe incidents. Here, the comparison between pediatric and adult patients with HOCM has been performed to uncover the signaling mechanism regulating pathological process through histopathological analysis and immunohistochemical analysis. We found SMAD proteins played an important role during myocardial fibrosis for HOCM patients. In patients with HOCM, Masson and HE staining showed that myocardial cells were diffusely hypertrophied with obvious disorganized myocardial fiber alignment, and myocardial tissue was more damaged and collagen fibers increased significantly, which come early in childhood. Increased expressions of SMAD2 and SMAD3 contributed to myocardial fibrosis in patients with HOCM, which happened early in childhood and continued through adulthood. In addition, decreased expression of SMAD7 was closely related to collagen deposition, which negatively expedited fibrotic responses in patients with HOCM. Our study indicated that the abnormal regulation of SMAD signaling pathway can lead to severe myocardial fibrosis in childhood and its fibrogenic effects persist into adulthood, which is a crucial factor in causing sudden cardiac death and heart failure in HOCM patients.

## Introduction

Sudden cardiac death and progressive heart failure are considered to be the most significant complications of hypertrophic obstructive cardiomyopathy (HOCM), while myocardial fibrosis is the main cause of lethal arrhythmias and heart failure in patients with HOCM^1^. Some studies confirmed the high burden of myocardial fibrosis in both young patients who suffered sudden cardiac death^2^ or older patients with HOCM suffering from advanced heart failure^3^. Our previous histopathological study demonstrated that pediatric patients with HOCM may present with severe myocardial fibrosis and reduced microvascular density^4^.

The transforming growth factor-β (TGF-β) superfamily proteins play a critical role in regulation of cardiac fibrotic responses, which is mediated via intracellular effectors (the Smads), or via activation of Smad-independent cascades^5,6^. The members of the Smad family are considered the best characterized intracellular effectors of TGF-β, which can be divided into three functional classes: (a). Receptor-activated Smads (R-Smads): including Smad1, Smad2, Smad3, Smad5 and Smad8, responsible for the TGF-β superfamily signaling pathway; (b). the co-mediator Smads (Co-Smads), the Smad4 forms a signaling complex with R-Smads; (c). the inhibitory Smads (I-Smads): Smad6, Smad7, involved in the negative regulation of the R-Smad-mediated cascades^7^. Extensive experimental evidences suggest fibroblast-specific deletion of *Smad2/3* from cardiac fibroblasts prevented the gene program for fibrosis and extracellular matrix (ECM) remodeling^8^. Activation of Smad2/3 cascade is critical in ECM gene expression and regulation of fibrous tissue deposition^9^. Smad3 is a key activating signal for cardiac fibroblast to function at inducing myofibroblast conversion, stimulating the transcription of extracellular matrix proteins, and promoting a matrix-preserving phenotype^10^. In vitro studies also suggest that Smad2 plays a significant role in activation of a fibrogenic transcriptional program^7^. Smad2 knockdown was proved to inhibit incorporation of α-SMA into myofibroblast stress fibers^9^. In contrast, Smad3 appears more important than Smad2 in mediating fibroblast activation and fibrosis in vivo^8^, Smad7 can negatively feedback block the activation of Smad2/Smad3, so the Smad7 has a protective role in MF and ventricular remodeling^11^. Aberrant Smad family expression can occur in a variety of cardiac pathophysiological states. Fibroblasts, vascular cells and cardiomyocytes, the major cellular effector cells of cardiac fibrosis, are highly responsive to TGF-β and play a critical role in regulating the fibrotic process by activating Smad-dependent signaling pathways.

In this study, we analyzed the expression levels of SMAD proteins in septal myocardial tissue samples from pediatric and adult patients with HOCM after Morrow procedure using immunohistopathological methods, focusing on the effect of SMAD signaling on myocardial fibrosis to further elucidate the evolution of myocardial fibrosis in patients HOCM.

## Materials and methods

### Study population

Pediatric and adult patients with hypertrophic obstructive cardiomyopathy who underwent transaortic septal myocardial resection (Morrow procedure) were selected from March 2004 to December 2018 at the heart center of the First Hospital of Tsinghua University. The control group was obtained from ventricular septal myocardial tissue of pediatric and adult autopsy individuals who died of non-cardiac disease. Diagnostic criteria for hypertrophic obstructive cardiomyopathy: left ventricular wall thickness, or septal thickness ≥ 15 mm, left ventricular outflow tract pressure difference at rest > 30 mmHg, or > 50 mmHg at provocation, and can exclude other causes of ventricular hypertrophy. In pediatric patients, the increase in LV wall thickness exceeded the mean LV wall thickness plus 2 standard deviations (or Z value > 2) for children of the same age, sex, and body surface area, and other conditions causing increased cardiac load were excluded. Exclusion criteria: coronary artery disease (> 50% stenosis on coronary angiography), uncontrollable hypertension (defined as blood pressure above 140/90 mmHg), valvular heart disease, infection, or renal insufficiency. Twelve adult and five pediatric patients with hypertrophic obstructive cardiomyopathy were included in this study. Control myocardium from the LV septal wall was collected at autopsy of five adults and four pediatric individuals, who died of non-cardiac causes. Clinical information was obtained from the hospital electronic medical record system (Table 1).

**Table 1:** Baseline characteristics of adult and pediatric patients with HOCM.

Variable	Adult with HOCM (n = 12)	Pediatric with HOCM (n = 5)
Age (years)	47.4 ± 7.9	9.30 ± 4.00
Male, n (%)	11 (92%)	2(40%)
BMI (kg/m^2^)	26.22 ± 4.16	18.60 ± 4.64
Symptoms		
Chest pain	5 (41.67%)	1 (20%)
Syncope	2 (16.67%)	
Dyspnea	8 (66.67%)	1 (20%)
Echocardiography		
LVOT PG at rest(mmHg)	96.8 ± 44.1	89.4 ± 29.4
Septal wall thickness (mm)	25.7 ± 9.4	16.2 ± 1.2
LV end-diastolic diameter (mm)	47.67 ± 7.46	22.60 ± 4.28
LVEF (%)	63.48 ± 5.29	72.80 ± 7.12
Family history of HCM n (%)	2 (16.67%)	
Medications, n (%)		
β-Blockers	8 (66.67%)	1 (20%)
Calcium channel blockers	3 (25%)	

### Echocardiography

Standard transthoracic M-mode, 2-dimensional, pulsed or continuous wave doppler images were acquired using a Philips IE33 color Doppler system (Philips Healthcare, Andover, MA, USA). Parameter acquisition was performed according to the American Society of Echocardiography guidelines^12^. The detection of peak velocity and differential pressure across the left ventricular outflow tract was calculated using the simplified Bernoulli equation. Left ventricular outflow tract obstruction was defined as hypertrophic cardiomyopathy diagnosed as left ventricular thickness or septal thickness ≥ 15 mm or left ventricular outflow tract differential pressure > 30 mmHg at rest or > 50 mmHg at provocation^13^.

### Histological analysis

The septal myocardium samples were fixed in 10% formalin, embedded in paraffin. The samples were cut into 5 μm sections and stained with hematoxylin and eosin (H&E) to analyze the morphology. The samples were also stained with Masson trichrome to analyze the extent of myocardial fibrosis^14^.

### Immunohistochemical staining

Septal myocardial tissue specimens were fixed in 10% buffered formalin, embedded in paraffin, and cut into 5um thick tissue sections for immunohistochemical staining. After deparaffinization and rehydration with graded concentrations of ethanol to deionized water, the sections were heated in EDTA antigen retrieval buffer (pH9.0, ZSGB-BIO) in a pressure cooker for 2.5 min for antigen retrieval. Endogenous peroxidase activity was bloked with 3% (vol/vol) H_2_O_2_. Then, the sections were incubated with 10% goat serum for 1 h at room temperature, followed by different primary antibodies at 4 °C overnight: anti-Smad2 (diluted 1:20, Abcam), or anti-Smad3 (diluted 1:500, Abcam), or anti-MADH7/SMAD7 (diluted 1:100, Abcam), or anti-Collagen I (diluted 1:1000, Abcam). The sections were subjected to horseradish peroxidase (HRP)-conjugated goat anti-rabbit IgG (diluted 1:1000, Abcam) for 1 h at room temperature and then visualized with 3,3-diaminobenzidine (DAB) solution (ZSGB-BIO). Finally, the sections were counterstained using hematoxylin.

Images were captured using Motic EasyScan 60 and analyzed with Image -pro plus software (Image Pro Plus 6.0; Media Cybernetics Inc. Media Cybernetics, Inc; 4340 East–West Hwy, Suite 400 Bethesda, MD; USA). Ten areas per section were randomly selected, and the images were magnified × 40 magnification, and the positive expression areas were brown in color. Image-Pro Plus 6.0 image analysis software was used to calculate the integrated optical density (IOD) value, IOD indicates the protein expression of each index, the higher the IOD value, the higher the protein expression.

### Statistical methods

Statistical data were analyzed using SPSS 25.0 (SPSS Inc., Chicago, IL, USA). Data were expressed as mean ± standard deviation. Continuous variables were compared between the two groups using an independent *t*-test. *p* values less than 0.05 were used as a test for significant differences.

### Ethics declarations

The study was supported by the Ethics Committee of the First Hospital of Tsinghua University, and informed consent was obtained from patients or legal guardians. All experiments were performed in accordance with relevant guidelines and regulations.

## Results

### Clinical baseline characteristics of patients with HOCM

Demographic and clinical baseline characteristics of the study population are presented in Table 1. The mean age of the 12 adult HOCM patients and 5 pediatric HOCM patients was 41.5 ± 15.4 years and 9.30 ± 4.00 years, respectively. The left ventricular outflow tract PG max (mmHg) was 96.8 ± 44.1 mmHg in the adult HOCM group and 89.4 ± 29.4 mmHg in the pediatric HOCM group.

### Changes in ventricular septal myocardial tissue and cell morphology in patients with HOCM

**Figure 1:**
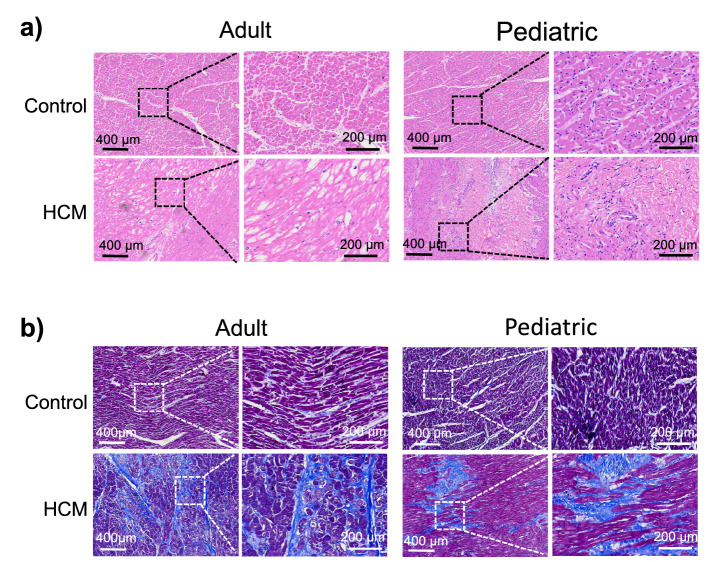
Morphological examinations of myocardial tissue from patients with HOCM. (**a**) Representative images of haematoxylin and eosin (H&E) staining of ventricular septal myocardial tissue from adult HOCM and pediatric HOCM patients, compared to their controls. Scale bars, 400 μm and 200 μm (enlarged views of boxed areas). (**b**) Representative images of Masson’s Trichrome staining of heart sections from adult HOCM and pediatric HOCM patients, compared to their controls. Scale bars, 400 μm and 200 μm (enlarged views of boxed areas).

HOCM is a genetic disorder characterized by thickened septum between the ventricles, which is usually accompanied by dynamic left ventricular outflow tract obstruction. First, we depicted the changes of cell gross morphology, muscle fiber arrangement, and organization of the architecture in the hearts with HOCM using histological analysis. HE staining showed that in the adult control and pediatric control groups, myocardial cells were structurally intact and well-arranged; however, in the adult HOCM and pediatric HOCM groups, myocardial cells were diffusely hypertrophied with obvious disorganized myocardial fiber alignment (Fig. 1a). Masson staining showed that myocardial fibrotic areas were blue and myocardial cells were red (Fig. 1b): myocardial tissue was neatly arranged and evenly stained with fewer collagen fibers in adult control and pediatric control groups; while myocardial tissue was more damaged and collagen fibers increased significantly in adult HOCM and pediatric HOCM patients.

Therefore, in patients with HOCM, myocardial cells were diffusely hypertrophied with obvious disorganized myocardial fiber alignment, and myocardial tissue was more damaged and collagen fibers increased significantly, which come early in childhood.

### Increased SMAD2 and SMAD3 expressions in ventricular septal myocardial tissue from patients with HOCM

**Figure 2:**
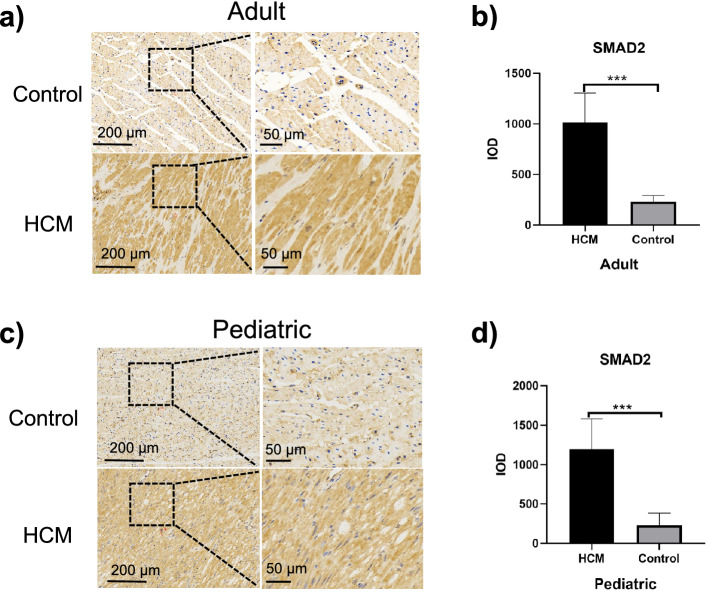
Immunohistochemical analysis of SMAD2 expression in ventricular septal myocardial tissue from patients with HOCM. (**a**) Representative images of immunohistochemical staining of SMAD2 in ventricular septal myocardial tissue from the control and adult HOCM patients. Scale bars, 200 μm and 50 μm (enlarged views of boxed areas). **b.** Quantification of SMAD2 expression level in ventricular septal myocardial tissue from the control and adult HOCM patients. Data are mean ± SEM, ***p < 0.001. **c.** Representative images of immunohistochemical staining of SMAD2 in ventricular septal myocardial tissue from the control and pediatric HOCM patients. Scale bars, 200 μm and 50 μm (enlarged views of boxed areas). **d.** Quantification of SMAD2 expression level in ventricular septal myocardial tissue from the control and pediatric HOCM patients. Data are mean ± SEM, ***p < 0.001.

**Figure 3:**
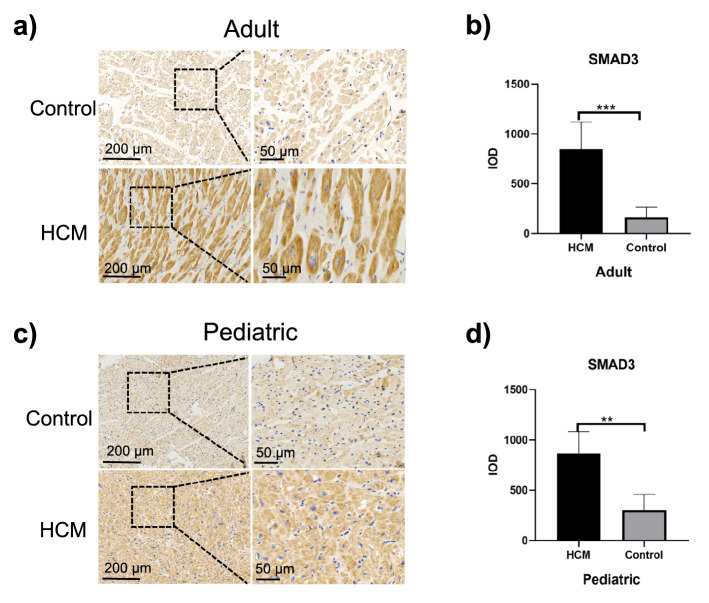
Immunohistochemical analysis of SMAD3 expression in ventricular septal myocardial tissue from patients with HOCM. (**a**) Representative images of immunohistochemical staining of SMAD3 in ventricular septal myocardial tissue from the control and adult HOCM patients. Scale bars, 200 μm and 50 μm (enlarged views of boxed areas). (**b**) Quantification of SMAD3 expression level in ventricular septal myocardial tissue from the control and adult HOCM patients. Data are mean ± SEM, ***p < 0.001. **c.** Representative images of immunohistochemical staining of SMAD3 in ventricular septal myocardial tissue from the control and pediatric HOCM patients. Scale bars, 200 μm and 50 μm (enlarged views of boxed areas). (**d**) Quantification of SMAD3 expression level in ventricular septal myocardial tissue from the control and pediatric HOCM patients. Data are mean ± SEM, **p < 0.01.

Given that increased fibrosis appeared in HOCM hearts, and TGF-β-Smad2/3 signaling is considered as principal mediators of the fibrotic response, Smad2 and Smad3 even serving as their major downstream effectors^15^, we hypothesized that expressions of SMAD2 and SMAD3 might be upregulated in patients with HOCM. Using immunohistochemical staining, we found expressions of SMAD2 and SMAD3 in myocardial tissue of adult HOCM patients were significantly higher than those of their controls (1014.00 ± 290.10 vs. 230.50 ± 60.91 *p* = 0.0001 Fig. 2a,b; 846.80 ± 274.30 vs. 162.40 ± 102.50 *p* = 0.0003 Fig. 3a,b). Similarly, expressions of SMAD2 and SMAD3 in myocardial tissue of pediatric with HOCM were significantly higher than those of their controls (1197.00 ± 383.80 vs. 229.00 ± 155.10 *p* = 0.0008 Fig. 2c,d; 867.30 ± 217.50 vs. 301.30 ± 158.31 *p* = 0.0015 Fig. 3c,d). In addition, there was no significant difference in expressions of SMAD2 and SMAD3 in myocardial tissues between adult HOCM patients and pediatric HOCM patients (Fig. 6a,b).

Thus, increased expressions of SMAD2 and SMAD3 contributed to myocardial fibrosis in patients with HOCM, which happened early in childhood and continued through adulthood.

### Decreased SMAD7 expression and collagen I deposition in ventricular septal myocardial tissue from patients with HOCM

**Figure 4:**
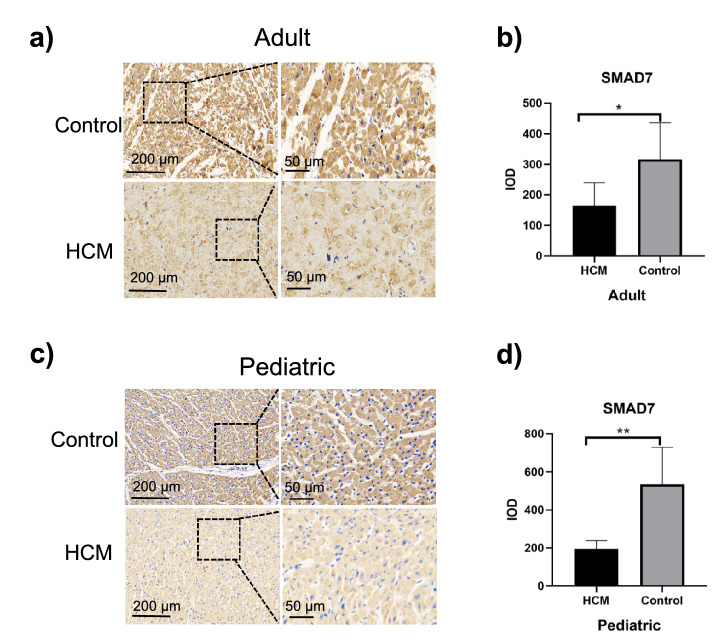
Immunohistochemical analysis of SMAD7 expression in ventricular septal myocardial tissue from patients with HOCM. (**a**) Representative images of immunohistochemical staining of SMAD7 in ventricular septal myocardial tissue from the control and adult HOCM patients. Scale bars, 200 μm and 50 μm (enlarged views of boxed areas). (**b**) Quantification of SMAD7 expression level in ventricular septal myocardial tissue from the control and adult HOCM patients. Data are mean ± SEM, *p < 0.05. (**c**) Representative images of immunohistochemical staining of SMAD7 in ventricular septal myocardial tissue from the control and pediatric HOCM patients. Scale bars, 200 μm and 50 μm (enlarged views of boxed areas). (**d**) Quantification of SMAD7 expression level in ventricular septal myocardial tissue from the control and pediatric HOCM patients. Data are mean ± SEM, **p < 0.01.

Smad7 is an important negative regulator of TGF-β/Smad signaling that may inhibit cardiac fibrosis, whose overexpression in cardiac fibroblasts restrains collagen synthesis^16^. In adult patients with HOCM, SMAD7 expression was remarkably reduced in ventricular septal myocardial tissue when compared to the control group (164.70 ± 76.11 vs. 316.70 ± 119.20 *p* = 0.0110 Fig. 4a,b), likewise, SMAD7 expression in myocardial tissue of pediatric patients with HOCM was obviously lower than that of their controls (195.10 ± 43.37 vs. 534.80 ± 195.00 *p* = 0.0052 Fig. 4c,d). In addition, there was no significant difference in the expression of Smad7 in myocardial tissues between adult HOCM patients and pediatric HOCM patients (Fig. 6c).

**Figure 5:**
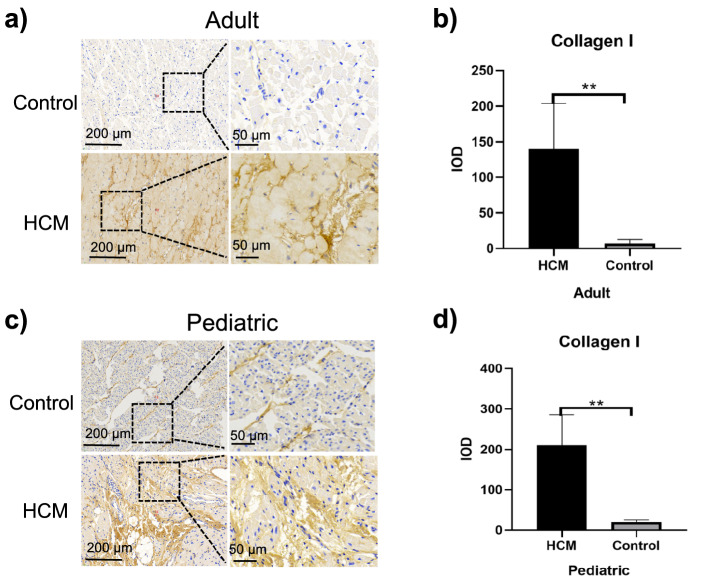
Immunohistochemical analysis of type I collagen expression in ventricular septal myocardial tissue from patients with HOCM. (**a**) Representative images of immunohistochemical staining of type I collagen in ventricular septal myocardial tissue from the control and adult HOCM patients. Scale bars, 200 μm and 50 μm (enlarged views of boxed areas). (**b**) Quantification of type I collagen expression level in ventricular septal myocardial tissue from the control and adult HOCM patients. Data are mean ± SEM, **p < 0.01. (**c**) Representative images of immunohistochemical staining of type I collagen in ventricular septal myocardial tissue from the control and pediatric HOCM patients. Scale bars, 200 μm and 50 μm (enlarged views of boxed areas). (**d**) Quantification of type I collagen expression level in ventricular septal myocardial tissue from the control and pediatric HOCM patients. Data are mean ± SEM, **p < 0.01.

**Figure 6:**
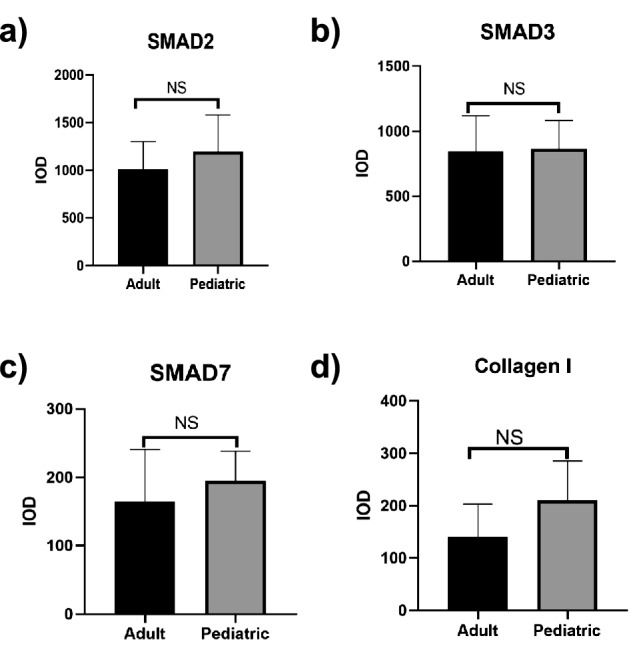
Comparison of expression levels of SMAD2, 3, 7, and type I collagen in adult and pediatric HOCM patients. (**a**) Comparison of the expression level of SMAD2 in adult and pediatric HOCM patients. Data are mean ± SEM, ns, non-significant, (*p* > 0.05). (**b**) Comparison of the expression level of SMAD3 in adult and pediatric HOCM patients. Data are mean ± SEM, ns, non-significant, (*p* > 0.05). (**c**) Comparison of the expression level of SMAD7 in adult and pediatric HOCM patients. Data are mean ± SEM, ns, non-significant, (*p* > 0.05). (**d**) Comparison of the expression level of type I collagen in adult and pediatric HOCM patients. Data are mean ± SEM, ns, non-significant, (*p* > 0.05).

Collagen is a major component of the myocardium^17^. Fibrosis is typically characterized by an accumulation of fibrillar collagens, especially of collagen type I^18^. The expression of type I collagen in myocardial tissues of adult HOCM patients was significantly increased when compared with the control group (139.80 ± 63.76 vs. 7.26 ± 5.26 *p* = 0.0039 Fig. 5a,b), and its expression in myocardial tissue of pediatric with HOCM dramatically higher than that of their controls (210.80 ± 74.92 vs. 20.41 ± 5.42 *p* = 0.0053 Fig. 5c,d). There was no significant difference in the expression of type I collagen in myocardial tissues between adult HOCM patients and pediatric HOCM patients (Fig. 6d).

Overall, besides of positive regulation to myocardial fibrosis by increased expression of SMAD2 and SMAD3, decreased expression of SMAD7 was closely related to collagen deposition, which negatively expedited fibrotic responses in patients with HOCM. As for HOCM, SMAD signaling may start as early as childhood.

## Discussion

This study demonstrated that significantly increased expression levels of SMAD2/3 and sharply reduced expression levels of SMAD7 occurred in ventricular septal myocardial tissue samples from pediatric and adult patients with HOCM who underwent Morrow surgery, while there were no significant differences between pediatric and adult patients. It indicates that abnormal regulation of SMAD signaling in HOCM patients may contribute to the progressive exacerbation of myocardial fibrosis and cause severe cardiac failure.

Smad proteins are the main downstream effector molecules in the TGF-β signaling pathway. Under certain pathophysiological conditions, the expression of TGF-β and its superfamily members is enhanced in myocardial tissue, which triggers a heterotetrameric complex formation through binding to their receptors, TGF-β receptor I (TβRI) or TGF-β receptor II (TβRII). Activated TβRI recruits and phosphorylates downstream receptor-regulated Smad proteins (Smad2 and Smad3), and activated Smad2 and Smad3 bind to Smad4 to form Smad complexes and enter the nucleus, initiating the expression of fibrosis-related transcription factors and proteins^7^. Clinical investigations suggested TGF-β1 expression were upregulated in myocardial tissue of patients with congenital hypertrophic cardiomyopathy^19^. Circulating TGF-β levels were elevated and associated with adverse cardiac events in patients with hypertrophic cardiomyopathy^20^. Smad activation in fibrotic cardiac condition are considered as the results of induction of TGF-β superfamily members^7^. Previous genetic-based studies demonstrated that canonical Smad2 and Smad3 signaling cascade were more dedicated to control fibrosis-mediating genes in activated fibroblast-specific *Tgfbr1/2* and *Smad2/3*-deleted mice^8^. *Smad2/3* deletion in mouse fibroblasts comprises TGF-β-induced fibrosis. In myocardial infarction studies, activation of Smad2 and Smad3 led to myocardial fibrosis and cardiac remodeling^21^. Smad3 stimulates transcription of structural and matrix extracellular matrix proteins, including type I and III collagen, fibronectin, and periostin, and induces synthesis of matrix cross-linking enzymes^8,22^. In contrast to the critical effects in mediating myofibroblast conversion and cardiac fibrosis, Smad3 function dominantly over Smad2. Smad2 plays an important role in regulation of the reparative response following cardiac remodeling of the infarcted heart^8^. Loss of Smad2 did not affect scar organization, but was associated with delayed dilative remodeling^21^. Although the Smad signaling cascade is well defined in regulation of fibrotic responses in murine hearts, the knowledge of its function in patients with HOCM is still limited. In this histopathological comparison of 12 adult and 5 pediatric patients with HOCM, we demonstrated that Smad2/3 protein expression was significantly increased in the ventricular septal tissue in HOCM patients with extensive myocardial fibrosis, which indicated that the Smad2/3 signaling cascade is a persistent stimulus for the development of hypertrophic cardiomyopathy. Thus, Smad2/3 induced myocardial fibrosis is an important cause of sudden cardiogenic death in pediatric patients and late heart failure in adults.

Smad7 serves as a negative regulator of TGF-β1/Smads signaling pathway, which binds to activated TβRI to inhibit the phosphorylation and activation of R-Smads and antagonize TGF-β1 signaling^23^. Smad7 activation acts as a TGF-β-induced endogenous inhibitory signal that prevent myocardial fibrosis. Decreased Smad7 expression results in cardiac fibrosis in the infarcted rat heart, whereas Smad7 overexpression in cardiac fibroblasts inhibits collagen synthesis^16,24^. In our study, we found that SMAD7 expression significantly reduced in myocardial tissue of pediatric and adults with HOCM while SMAD2/3 expression increased, which is consistent with a previous report that Smad7 loss accentuated Smad2/3 activation^25^. Thus, with respect to patients with HOCM, the steady state controlled by the antifibrotic effects of SMAD7 and the fibrotic responses of SMAD2/3 might be disturbed.

The characteristic pathophysiological changes in hypertrophic cardiomyopathy are myocardial cell abnormalities, disorganized arrangement and interstitial fibrosis. The main components of the extracellular matrix (ECM) are collagen and glycosaminoglycans (GAGs)^26^. In hypertrophic cardiomyopathy, the most typical ECM components are collagens I and III, and the collagen content is heavily aggregated, affecting cardiac diastolic function^17,27^. In this study, we found that myocardial tissue collagen I expression was significantly elevated in pediatric and adults with HOCM, while SMAD2 and SMAD3 increased and inhibitory SMAD7 decreased in myocardial tissue, which was closely related to myocardial fibrosis described above. Therefore, activated SMAD signaling may be the direct cause of the fibrotic outcome of HOCM. And in our previous study, we found that patients with HOCM had reduced microvascular density and impaired myocardial perfusion^4^. The main role of MMP is to degrade type I and III collagen, and its elevated activity can cause increased fibrillar collagen degradation, extracellular matrix remodeling, and ventricular dilation^28^. Bi et al. found in patients with HOCM that myocardial MMP-2/TIMP-1 ratio was elevated by reduced microvasculature and that high plasma PICP/ICTP and MMP-2/TIMP-1 ratio were independent predictors of adverse outcomes in patients with HOCM^29^. So, our results have extended the understanding of the mechanism of HOCM, which helps to explore the new therapeutic treatments for HOCM patients as early as possible.

To date, more and more important molecules and crosstalk involved in the development of HOCM were revealed by bioinformatics technology, such as lncRNAs (XIST, MALAT1, and H19), TFs (SPI1 and SP1), and miRNAs (has-miR-29b-39 and has-miR-29a-3p)^30^. Transcription factors (TFs) can control the molecular network that regulates cardiomyocyte hypertrophy. Among them, SP1 is able to affect cardiomyocyte hypertrophy by SP1/GATA4 signaling pathways^30^, MRTF-A-Sp1-PDE5 axis^31^, and ROCK1-Sp1-PKCγ axis^32^. The study earlier reported that SP1 plays a crucial role in the pathogenesis and the progression of human glomerulonephritis probably via cooperation with pSmad2/3 and p300^33^. More importantly, many of the functions of TGF-β are mediated through Sp1 in cooperation with Smad signaling^34^. p300 is a transcriptional co-activator that may be involved in the activation of Smad complexes^35^. So, in our further research, Sp1 may be an important candidate that help uncover the more complicated pathogenic mechanism of HOCM.

## Limitations

This study also has some limitations. As a retrospective study, the research methods restricted to the sample size and sample types (only apply to the relevant examinations). First, the number of cases in this manuscript is slightly less, particularly the samples from pediatric patients with HOCM are very rare, but they are crucial to understand the development and mechanism of HOCM. Second, formalin/PFA- fixed paraffin-embedded tissues are long-lasting and easy to archive, but these are only fit for immunohistochemical investigations, which limits its applications. For these reasons, we will try to use some advanced technologies to the small but rare sample, such as using single cell RNA sequencing (scRNA-seq) to display comprehensive gene expression profiling of different cell types from the HOCM myocardial tissue, and to reveal the gene regulatory network or the crosstalk among different cell types by combining with bioinformatics technology. Also, we will collect RNA samples and protein samples from fresh tissues for data validations. Further studies with multi-level confirmations using various sample types, correlations between gene or transcript levels, and clinical prognosis will be carried out in the next part of our plans.

## Conclusions

In myocardial tissue from patients with HOCM, SMAD2/3 and type I collagen expression levels are elevated, whereas inhibitory SMAD7 protein expression levels are reduced, and their expression levels do not differ significantly between pediatric and adult patients with HOCM. These indicate that myocardial fibrosis due to SMAD signaling pathway activation occurs in childhood and that its fibrogenic effects persist and are a crucial factor in causing sudden cardiac death and heart failure in HOCM patients.

## Data Availability

All data related to this manuscript are available from the corresponding author upon reasonable request.

## Abbreviations

HOCM: Hypertrophic obstructive cardiomyopathy
TGF-β: The transforming growth factor-β
H&E: Hematoxylin and eosin
HF: Heart failure
R-Smads: Receptor-activated Smads
Co-Smads: Co-mediator Smads
I-Smads: Inhibitory Smads
LV: Left ventricular
IOD: Integrated optical density
α-SMA: α-Smooth muscle actin
MMP: Matrix metalloproteinase
ECM: Extracellular matrix
GAGs: Glycosaminoglycans
scRNA-seq: Single cell RNA sequencing
PICP/ICTP: Carboxy-terminal propeptide of procollagen type I/carboxy-terminal telopeptide procollagen type I
